# Effect of I-PRF and alloplastic bone material in periodontal osseous defects - A case series

**DOI:** 10.6026/973206300214634

**Published:** 2025-12-15

**Authors:** Kriti Agarwal, Kanika Agarwal, Jaideep Mahendra, Roshini Govindarajan, Sajid T Hussain, Muskan Bedi

**Affiliations:** 1 Department of Periodontology, Santosh Dental College, Ghaziabad, Uttar Pradesh, India; 2 Private Practitioner, Prayagraj, Uttar Pradesh, India; 3 Department of Periodontics, Director of Postgraduate studies, Meenakshi Ammal Dental College and Hospital, Chennai, Tamil Nadu, India; 4 Department of Periodontics, Meenakshi Ammal Dental College and Hospital, Chennai, Tamil Nadu, India; 5 Department of Periodontology and Implantology, Bharath University, Sree Balaji Dental College and Hospital, Pallikaranai, Chennai, Tamil Nadu, India; 6 Department of Surgery, Sree Ramachandra Medical College and Research Institute, Porur, Chennai, Tamil Nadu, India

**Keywords:** Chronic periodontitis, Hydroxyapatite, Intraosseous defect, i-PRF, periodontal regeneration

## Abstract

Periodontal regeneration in patients with chronic periodontitis remains a clinical challenge due to limited predictability of current regenerative materials and techniques. This case series was conducted on 10 patients diagnosed with moderate to advanced chronic periodontitis. Participants received i-PRF with Hydroxyapatite bone graft. Clinical parameters and radiographic bone defect depth were measured at baseline and after 6 months. Following surgical intervention, patients exhibited greater reductions in PPD, gains in CAL and increased bone fill suggesting that i-PRF may enhance periodontal regeneration when used in conjunction with alloplastic bone grafts.

## Background:

Regeneration is the natural renewal of a structure by growth and differentiation of new cells and intercellular substances to form new tissues or parts [1]. Periodontal regeneration is healing after periodontal surgeries that result in the formation of new attachment apparatus, consisting of cementum, periodontal ligament and alveolar bone [2]. A number of innovative treatment modalities using various bone grafts, Guided Tissue Regeneration, biological mediators and growth factors have been used to promote periodontal regeneration [3]. Substantial variations in clinical predictability, degree of efficacy of this reaction have been reported which depends on the type of morphologic defect surgical precision and patient's post-operative maintenance [4]. The application of growth factors or biological response molecules to stimulate cells responsible for periodontal regeneration has been proposed [5]. Growth factors is a general term used to denote a class of naturally occurring proteins that function in the body to promote the mitogenesis directed migration and metabolic activity of the cells [6]. Growth factors are released at low concentrations over short intercellular distances, where they bind to high-affinity membrane receptors that activate second-messenger pathways and gene expression, thereby regulating key cellular processes such as proliferation, differentiation and the synthesis of additional mediators including platelet-derived, transforming, fibroblast-derived and vascular endothelial growth factors [7]. In recent times, autologous platelet concentrates have shown great promise in periodontal regeneration, as they provide a biologically active source of growth factors, with platelet-rich plasma offering a high concentration of platelets and associated regenerative mediators that enhance the effect of bone grafts [8]. Platelet-rich fibrin (PRF), a second-generation autologous platelet concentrate, forms a leukocyte- and fibrin-rich matrix, and its injectable form (i-PRF) contains a slowly polymerizing fibrin network enriched with cytokines, glycoproteins and key regenerative mediators including pro- and anti-inflammatory cytokines (IL-1β, IL-6, TNF-α, IL-4), VEGF, FGF, angiopoietin and PDGF, that collectively enhance tissue healing [9, 10]. By localizing growth factors and increasing their concentration at the target site, i-PRF through its fibrin-rich network that enhances cell migration, proliferation, and the transport of regenerative cells and has demonstrated beneficial effects across various procedures, including facial plastic surgery, sinus lift augmentation, use as an osteoconductive filling material, and the treatment of multiple gingival recession defects [11]. The injectable form of i-PRF enables precise delivery into periodontal pockets, and research shows that when applied in repeated sessions, it produces superior regenerative results during the first six months, particularly in terms of defect fill and resolution [12]. When combined with bone graft materials, i-PRF has been shown to enhance the healing response, often resulting in greater defect fill and measurable reductions in probing depth [13]. As recent trends suggest that autologous platelet concentrates hold significant potential for regenerative applications in Periodontics, this case series explored their use in clinical practice. Therefore, it is of interest to examine the clinical effectiveness of autologous i-PRF when combined with alloplastic bone graft material in the treatment of periodontal osseous defects.

## Materials and Methodology:

## Patient selection:

This case series was conducted in accordance with institutional ethical guidelines and was approved by the Ethical Committee of Meenakshi Ammal Dental College and Hospital, Chennai. A total of 10 systemically healthy patients, aged 25 to 50 years and diagnosed with moderate to advanced chronic periodontitis, who presented for treatment at the Department of Periodontology, were included in this case series. Before treatment, informed consent was obtained from all the patients after receiving detailed information about the procedure.

## Inclusion criteria were:

presence of moderate to severe periodontitis, interproximal angular bone defects ≥3 mm in depth (radiographically), probing depth ≥5 mm and clinical attachment loss ≥5 mm. Exclusion criteria included systemic diseases, smoking, aggressive periodontitis, pregnancy or lactation and teeth with hopeless prognosis. Each patient underwent a thorough clinical and radiographic examination, including a detailed medical and dental history.

## Clinical parameters and measurements:

The following clinical indices were recorded at baseline and at 6 months postoperatively: Plaque Index (PI), Gingival Index (GI), Probing Pocket Depth (PPD), Clinical Attachment Level (CAL) and Radiographic Defect Depth (DD) [14, 15].

## Measurements recorded:

The following measurements were recorded at baseline and at 6 months postoperatively: Reference point (RP) to Gingival Margin (GM), RP to Cementoenamel Junction (CEJ) and RP to Base of the Pocket (BOP).

## Radiographic assessment:

Standardized intraoral periapical radiographs were taken at baseline and at 6 months to assess the radiographic defect depth (DD), using the long cone paralleling technique. All patients demonstrated vertical/angular bone defects on radiographs. The percentage of bone fill was calculated at baseline and 6 months [16].

## Presurgical phase:

All 10 patients presenting with periodontal osseous defects underwent an initial evaluation including diagnosis and treatment planning. Phase I periodontal therapy included full-mouth supragingival and subgingival scaling and root planing, along with personalized oral hygiene instructions. After a 4-week healing period, patients were re-evaluated. Those demonstrating satisfactory tissue response and compliance with plaque control were considered for surgical intervention.

## Surgical phase:

## Surgical procedure:

Local anaesthesia was administered using 2% lignocaine with 1:80,000 adrenaline via infiltration. A sulcular incision was made using a #15 Bard-Parker blade, extending one tooth mesially and distally from the involved site. Vertical releasing incisions were given where required for better access. A full-thickness mucoperiosteal flap was elevated, and granulation tissue was debrided. Root surfaces were thoroughly scaled and planed using hand instruments. Defect morphology was confirmed intraoperatively and classified based on the number of remaining bony walls. Patients with treated with a combination of i-PRF and hydroxyapatite bone graft (Sybograf®, Eucare Pharmaceuticals Pvt. Ltd; particle size: 40-50 nm). i-PRF was prepared into a fibrin membrane by squeezing the aspirated i-PRF in sterile gauze [17]. The membrane was adapted at the base and over the graft material for enhanced stability and healing. Care was taken to avoid overfilling the defect. Interrupted 4-0 silk sutures (Ethicon, Johnson & Johnson, India) were used to achieve primary flap closure. A periodontal dressing was placed to protect the surgical site.

## Postoperative care:

Postoperative instructions included the administration of Amoxicillin 500 mg three times daily for 7 days and Ibuprofen 400 mg three times daily for 5 days and appropriate post-surgical instructions were provided.

## Maintenance phase:

Patients were recalled after one week for postoperative evaluation and to assess healing. Follow-up visits were conducted at 1, 3 and 6 months postoperatively for supportive periodontal therapy. At the 6-month visit, Plaque Index and Gingival Index were recorded and radiographs were taken to confirm the bone fill.

## Results and Discussion:

At baseline, patients exhibited deep periodontal pockets, clinical attachment loss and radiographic evidence of vertical bone defects. Following surgical intervention, progressive clinical improvements were noted across all cases. Patients demonstrated a clear reduction in probing pocket depth and appreciable gains in clinical attachment levels. Gingival tissues remained stable throughout the healing phase and no postoperative complications or adverse tissue responses were observed. Radiographic evaluation revealed consistent evidence of bone fill within the treated osseous defects. At the 6-month follow-up, all patients maintained adequate plaque control. Clinical examination confirmed substantial pocket depth reduction and attachment level gain, while radiographs demonstrated near-complete resolution of osseous defects in the majority of cases. Overall, the adjunctive application of i-PRF with hydroxyapatite bone graft yielded favourable regenerative outcomes in the management of periodontal osseous defects, particularly with respect to pocket depth reduction, clinical attachment gain and radiographic bone defect fill. The ultimate goal of periodontal therapy is to restore the periodontium by regenerating both bone and connective tissue attachment destroyed due to periodontal disease. This has led to the development and use of several growth factors such as insulin-like growth factors, fibroblast growth factors, platelet-derived growth factor (PDGF), transforming growth factor-beta (TGF-β), bone morphogenic proteins and others which promote cellular proliferation and tissue healing [18]. Platelet-rich fibrin (PRF), a second-generation platelet concentrate, acts as a vehicle carrying essential cells and growth factors necessary for tissue regeneration. Injectable PRF (i-PRF) releases critical growth factors like PDGF and TGF-β, which enhance healing and regeneration potential [12]. The present case series aimed to evaluate the clinical and radiographic efficacy of i-PRF with hydroxyapatite bone graft in the treatment of periodontal osseous defects over six months. This case series enrolled 10 systemically healthy patients with chronic periodontitis having vertical/angular bone defects. Patients were treated with a combination of i-PRF and hydroxyapatite bone graft. Baseline parameters such as plaque index (PI), gingival index (GI), probing pocket depth (PPD), clinical attachment level (CAL) and radiographic defect depth were measured. At six months post-operatively, all cases exhibited significant improvements in probing pocket depth (PPD) reduction, clinical attachment level (CAL) gain and radiographic bone fill, demonstrating notable clinical and radiographic outcomes. These findings were consistent with previous reports by Farimani *et al.* (2023) who observed similar outcomes at a 9-month follow-up [19]. The positive clinical response in this group can be attributed to the unique biological properties of i-PRF, which comprises a dense fibrin network enriched with platelets, leukocytes, cytokines, and circulating stem cells. Radiographically, all the cases showed considerable bone defect fill at six months. The results were in accordance with the results from Alshoiby *et al.* (2023) [20]. However, no significant differences were observed in plaque and gingival indices, which could be attributed to effective oral hygiene reinforcement throughout the study. The maintenance of low PI and GI scores in this case series indicates that the patients adhered well to plaque control measures. These findings differ from other studies like those of Chaudhary *et al.* (2022), who reported significant changes in PI and GI over longer follow-ups [20]. The clinical benefits observed can be attributed to the unique regenerative properties of i-PRF. Its dense fibrin network, enriched with platelets, leukocytes, cytokines and circulating stem cells, serves as a biologically active scaffold. This structure enables the gradual release of key growth factors such as TGF-β, PDGF-AB and VEGF, which play essential roles in angiogenesis, immune modulation, and matrix remodelling, osteoconduction and cell proliferation. This multifactorial action supports both soft and hard tissue regeneration, reinforcing the utility of i-PRF in periodontal therapy.

## Conclusion:

The significant use of i-PRF with hydroxyapatite bone graft, demonstrating notable improvements in bone fill and clinical parameters, thereby encouraging positive clinical and radiographic outcomes in the treatment of periodontal intrabony defectsn is discussed. The biologically active properties of i-PRF along with the combination treatment with hydroxyapatite bone graft, contribute to enhanced soft and hard tissue healing, resulting in improved pocket depth reduction, attachment gain and bone defect fill. Data support its use as a valuable tool in periodontal regenerative therapy.
